# Social Media Monitoring of the COVID-19 Pandemic and Influenza Epidemic With Adaptation for Informal Language in Arabic Twitter Data: Qualitative Study

**DOI:** 10.2196/27670

**Published:** 2021-09-17

**Authors:** Lama Alsudias, Paul Rayson

**Affiliations:** 1 Information Technology Department College of Computer and Information Sciences King Saud University Riyadh Saudi Arabia; 2 School of Computing and Communications Lancaster University Lancaster United Kingdom

**Keywords:** Arabic, COVID-19, infectious disease, influenza, infodemiology, infoveillance, social listening, informal language, multilabel classification, natural language processing, named entity recognition, Twitter

## Abstract

**Background:**

Twitter is a real-time messaging platform widely used by people and organizations to share information on many topics. Systematic monitoring of social media posts (infodemiology or infoveillance) could be useful to detect misinformation outbreaks as well as to reduce reporting lag time and to provide an independent complementary source of data compared with traditional surveillance approaches. However, such an analysis is currently not possible in the Arabic-speaking world owing to a lack of basic building blocks for research and dialectal variation.

**Objective:**

We collected around 4000 Arabic tweets related to COVID-19 and influenza. We cleaned and labeled the tweets relative to the Arabic Infectious Diseases Ontology, which includes nonstandard terminology, as well as 11 core concepts and 21 relations. The aim of this study was to analyze Arabic tweets to estimate their usefulness for health surveillance, understand the impact of the informal terms in the analysis, show the effect of deep learning methods in the classification process, and identify the locations where the infection is spreading.

**Methods:**

We applied the following multilabel classification techniques: binary relevance, classifier chains, label power set, adapted algorithm (multilabel adapted k-nearest neighbors [MLKNN]), support vector machine with naive Bayes features (NBSVM), bidirectional encoder representations from transformers (BERT), and AraBERT (transformer-based model for Arabic language understanding) to identify tweets appearing to be from infected individuals. We also used named entity recognition to predict the place names mentioned in the tweets.

**Results:**

We achieved an F1 score of up to 88% in the influenza case study and 94% in the COVID-19 one. Adapting for nonstandard terminology and informal language helped to improve accuracy by as much as 15%, with an average improvement of 8%. Deep learning methods achieved an F1 score of up to 94% during the classifying process. Our geolocation detection algorithm had an average accuracy of 54% for predicting the location of users according to tweet content.

**Conclusions:**

This study identified two Arabic social media data sets for monitoring tweets related to influenza and COVID-19. It demonstrated the importance of including informal terms, which are regularly used by social media users, in the analysis. It also proved that BERT achieves good results when used with new terms in COVID-19 tweets. Finally, the tweet content may contain useful information to determine the location of disease spread.

## Introduction

### Background

Although millions of items of data appear every day on social media, artificial intelligence through natural language processing (NLP) and machine learning (ML) algorithms offers the chance to automate their analysis across many different areas, including health. In the area of health informatics and text mining, social media data, such as Twitter data, can be analyzed to calculate large-scale estimates of the number of infections and the spread of diseases, or help to predict epidemic events [1]; this field is known as infodemiology, and the systematic monitoring of social media posts and Internet information for public health purposes is known as infoveillance. However, previous research has focused almost exclusively on English data.

Time is clearly an important factor in the health surveillance domain. In other words, discovering infectious diseases as quickly as possible is beneficial for many organizations and populations, as we have seen internationally with COVID-19. It is also important to have multiple independent sources to corroborate evidence of the spread of infectious diseases.

Twitter is one of the main real-time platforms that can be used in health monitoring. However, it contains noisy and unrelated information; hence, there is a crucial need for information gathering, preprocessing, and filtering techniques to discard irrelevant information while retaining useful information. One key task is to differentiate between tweets written for different reasons where someone is infected or worried about a disease, taking into account the figurative usage of some words related to a disease or spread of infection [2].

While such tasks are obviously relevant globally, there is little previous research for Arabic-speaking countries. There are some characteristics of the Arabic language that make it more difficult to analyze compared with other languages, and NLP resources and methods are less well developed for Arabic than for English. Arabic, which has more than 26 dialects, is spoken by more than 400 million people around the world [3]. We hypothesize that Arabic speakers will use their own dialects in informal discourse when they express their pain, concerns, and feelings rather than using modern standard Arabic [4]. Table 1 describes some examples of Arabic words related to health that may represent different meanings owing to dialect differences. For instance, the word 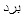 can be understood as influenza in Najdi dialect and feeling cold in Hejazi dialect [3].

**Table 1:** Some examples of Arabic words that have different meaning.

Word in Arabic	Potential meaning confusion sets
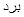	Influenza (cold)/feeling cold
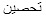	Vaccination/reading supplication
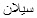	Runny nose/nosebleed
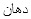	Ointment/paint
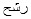	Sneezing (cold)/filter the liquid thing/be nominated for a position
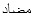	Antibiotic/opposite
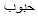	Tablets/pimples/some kind of food
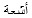	X-ray/sunlight
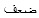	Weakness/double
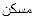	Painkiller/home
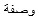	Prescription/method
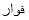	Medicine (like vitamin C fizz)/sparkling spring (fizz)

The real-world motivation of this work is to reduce the lag time and increase accuracy in detecting mentions of infectious diseases in order to support professional organizations in decreasing the spread, planning for medicine roll out, and increasing awareness in the general population. We also wish to show that Arabic tweets on Twitter can provide valuable data that may be used in the area of health monitoring by using informal, nonstandard, and dialectal language, which represents social media usage more accurately.

We focused on COVID-19 and influenza in particular owing to their rapid spread during seasonal epidemics or pandemics in the Arabic-speaking world and beyond. Most people recover within a week or two. However, young children, elderly people, and those with other serious underlying health conditions may experience severe complications, including infection, pneumonia, and death [5]. While it takes specialized medical knowledge to distinguish between the people infected by COVID-19 and influenza as the symptoms are similar, tracing and planning vaccination and isolation are important for both diseases. In addition, there may be some infected people who do not take the test because of personal concerns and lack of availability of tests in their city, or those who need support to self-isolate.

The overall question being answered in this paper is how NLP can improve the analysis of the spread of infectious diseases via social media. Our first main contribution is the creation of a new Arabic Twitter data set related to COVID-19 and influenza, which was labeled with 12 classes, including 11 originating from the Arabic Infectious Disease Ontology [6] and a new infection category. We used this ontology since there are no existing medical ontologies, such as International Classification of Diseases (ICD) and/or Systematized Nomenclature of Medicine-Clinical Terms (SNOMED), available that originate in Arabic [1]. Crucially, we also showed for the first time the usefulness of informal nonstandard disease-related terms using a multilabel classification methodology to find personal tweets related to COVID-19 or influenza in Arabic. We comparatively evaluated our results with and without the informal terms and showed the impact of including such terms in our study. Moreover, we showed the power of ML and deep learning algorithms in the classification process. Finally, we developed methods to identify the locations of the infectious disease spread using tweet content, and this also helped to inform dialect variants and choices.

### Related Work

Previous studies have proven that NLP techniques can be used to analyze tweets for monitoring public health [7-12]. These studies have analyzed social media articles that support the surveillance of diseases in different languages such as Japanese, Chinese, and English. Diseases that were analyzed included listeria, influenza, swine flu, measles, meningitis, and others. As justified in the previous section, we will focus on previous work related to monitoring influenza and COVID-19 using Twitter data.

#### Influenza-Related Research

The Ailment Topic Aspect Model (ATAM) is a model designed by Paul and Dredze [13]. It uses Twitter messages to measure influenza rates in the United States. It was later extended to consider over a dozen ailments and apply several tasks such as syndromic surveillance and geographical disease monitoring. Similarly, an influenza corpus was created from Twitter [8]. The tweets needed to meet the following two conditions to include them in the training data with infected people and timing: (1) the person tweeting or a close contact is infected with the flu and (2) the tense should be the present tense or recent past tense.

The goal of a previous study [2] was to distinguish between flu tweets from infected individuals and others worried about infection in order to improve influenza surveillance. It applied multiple features in a supervised learning framework to find tweets indicating flu. Likewise, a sentiment analysis approach was used [14] to classify tweets that included 12 diseases, including influenza. A forecasting word model was designed [15] using several words, such as symptoms, that appear in tweets before epidemics to predict the number of patients infected with influenza.

A previous study [16] used unsupervised methods based on word embeddings to classify health-related tweets. The method achieved an accuracy of 87.1% for the classification of tweets being related or unrelated to a topic. Another study [17] concluded that there is a high correlation between flu tweets and Google Trends data.

A recent survey study [1] showed how ontologies may be useful in collecting data owing to the structured information they contain. However, there were serious challenges as medical ontologies may consist of medical terms, while the text itself may contain slang terms. The study suggested the inclusion of informal language from social media in the analysis process in order to improve the quality of epidemic intelligence in the future, but this was not implemented.

#### COVID-19–Related Research

Many researchers in computer science have made extensive efforts to show how they can help during pandemics. In terms of NLP and social media, there are various studies that support different languages with multiple goals. These goals include defining topics discussed in social media, detecting fake news, analyzing sentiments of tweets, and predicting the number of cases [18].

There have been multiple Arabic data sets published recently [19,20]. The authors explained the ways of collecting tweets, such as time period, keywords, and software library used in the search process, and summed up the statistics for the collected tweets. However, they only included statistical analysis and clustering to generate summaries with some suggestion of future work. Yet, there are some studies with specific goals, such as analysis of the reaction of citizens during a pandemic [21] and identification of the most frequent unigrams, bigrams, and trigrams of tweets related to COVID-19 [22]. In addition, considering the study by Alanazi et al [23] that identified the symptoms of COVID-19 from Arabic tweets, the authors noted the limitation that they used modern standard Arabic keywords only, and it would be important to consider dialectical keywords in order to better catch tweets on COVID-19 symptoms written in Arabic, because some Arab users post on social media in their own local dialect.

In a previous study, we analyzed COVID-19 tweets in the following three different ways: (1) identifying the topics discussed during the period, (2) detecting rumors, and (3) predicting the source of the tweets in order to investigate reliability and trust [24].

Critically, none of the above studies utilized the Arabic language for monitoring the spread of diseases. There are some Arabic studies that used Twitter with the goal of determining the correctness of health information [25], analyzing health services [26], and proving that Twitter is used by health professionals [27]. Moreover, other studies, which did not involve Arabic, used only formal language terminologies when collecting tweets, and we would argue that this is not representative of the language usage in social media posts.

### Arabic Named Entity Recognition–Related Research

Previous research on named entity recognition (NER) aimed to accomplish the following two key goals: (1) the identification of named entities and (2) the classification of these entities, usually into coarse-grained categories, including personal names (PER), organizations (ORG), locations (LOC), and dates and times (DATE). In this study, our interest was in estimating one of these categories, which is the location element of the information on Twitter. NER methods use a variety of approaches, including rule-based, ML-based, deep learning–based, and hybrid approaches. These approaches can be used for Arabic, although specific issues arise, such as lack of capitalization, nominal confusability, agglutination, and absence of short vowels [28,29]. In addition, there are more challenges in terms of social media content, which includes Arabic dialects and informal terms. There is a lack of annotated data for NER in dialects. The application of NLP tools, originally designed for modern standard Arabic, on dialects leads to considerably less efficiency, and hence, we see the need to develop resources and tools specifically for Arabic dialects [29].

The goal of a previous study [30] was to illustrate a new approach for the geolocation of Arabic and English language tweets based on content by collecting contextual tweets. It proved that only 0.70% of users actually use the function of geospatial tagging of their own tweets; thus, other information should be used instead.

### Data Collection and Filtering

There is a lack of an available and reliable Twitter corpora in Arabic in the health domain, which makes it necessary for us to create our own corpus. We obtained the data using the Twitter application programming interface (API) for the period between September 2019 and October 2020, and collected around 6 million tweets that contained influenza or COVID-19 keywords. The keywords are in the code that we will release on GitHub [31]. We collected the tweets weekly since the Twitter API does not otherwise allow us to retrieve enough historical tweets. We utilized keywords related to influenza and COVID-19 from the Arabic Infectious Diseases Ontology [6], which includes nonstandard terminology. We used a disease ontology because it has been shown to help in finding all the terms and synonyms related to the disease [14].

A previous survey [1] suggested the inclusion of informal text used in social media in medical ontologies and search processes when collecting data in order to improve the quality of epidemic intelligence. Therefore, we hypothesize that informal terms may help to find the relevant tweets related to diseases. Additionally, in the Arabic scenario, we hypothesize that we need to account for dialectal terms.

We filtered the tweets by excluding duplicates, advertisements, and spam. Using Python, we also cleaned the tweets by removing symbols, links, non-Arabic words, URLs, mentions, hashtags, numbers, and repeating characters. From the resulting data set, we took a sample of about 4000 unique tweets (2000 tweets on influenza and 2000 tweets on COVID-19). Then, we used a suite of approaches for preprocessing the tweets, applying the following processes in sequence: tokenization, normalization, and stop-word removal. Table 2 shows the number of tweets with each label from the ontology after filtering and preprocessing.

**Table 2:** The number of tweets in each label.

Label	Tweets^a^, n	
	Influenza	COVID-19
Name of the disease	1544	1795
Slang term of the disease	456	327
Symptom	398	789
Cause	178	530
Prevention	666	209
Infection	51	15
Organ	2	202
Treatment	152	97
Diagnosis	25	2
Place of the disease spread	17	415
Infected category	52	12
Infected with	907	915

^a^Each tweet can have multiple labels.

### Manual Coding

In order to create a gold standard corpus, our process started with tweet labeling by two Arabic native speakers, including the first author of the paper, following the guidelines of the annotation process described in Multimedia Appendix 1. We manually annotated each tweet with 1 or 0 to indicate Arabic Infectious Diseases Ontology classes, which are infectious disease name (ie, influenza and COVID-19 in our case), slang term, symptom, cause, prevention, infection, organ, treatment, diagnosis, place of disease spread, and infected category. We also labeled each tweet as 1 if the person who wrote the tweet was infected with influenza or COVID-19 and 0 if not. Table 3 describes some examples of Arabic influenza and COVID-19 tweets with their labels.

**Table 3:** Examples of tweets with their assigned labels (1 or 0).

Tweet in Arabic	Tweet in English	Name	Slang name	Symptom	Cause	Prevention	Infection	Organ	Treatment	Diagnosis	Place of disease spread	Infected category	Infected with
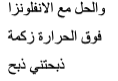	What is the solution with flu, fever and cold killed me	1^a^	1	1	0	0	0	0	0	0	0	0	1^b^
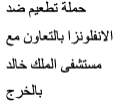	Influenza vaccination campaign in cooperation with King Khalid Hospital in Al-Kharj	1	0	0	0	1	0	0	0	0	0	0	0
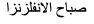	Flu morning	1	0	0	0	0	0	0	0	0	0	0	1
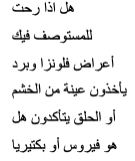	When you have symptoms of a flu or cold, Does the clinic take a sample of nose and throat to check if its bacteria or a virus	0	1	1	1	0	0	1	0	1	0	0	0
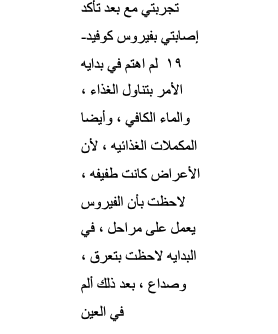	My experience with after my infection with the Covid-19 virus was confirmed, I did not initially care about eating food, enough water, and also food supplements, because the symptoms were slight, I noticed that the virus works in stages, at first I noticed sweating, headache, and then eye pain.	1	0	1	1	0	0	1	0	0	0	0	1
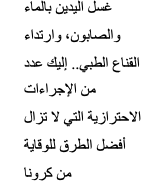	Washing hands with soap and water, and wearing a medical mask ... Here are a number of precautionary measures that are still the best ways to prevent Corona	0	1	0	0	1	0	0	0	0	0	0	0
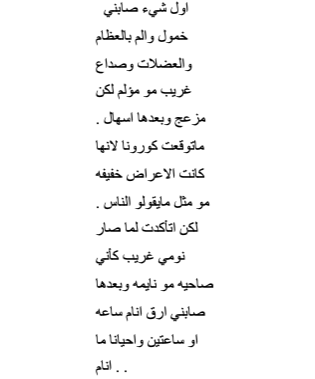	The first thing that struck me was lethargy, pain in the bones and muscles, a strange headache that was not painful but bothersome, and then had diarrhea. I did not expect Corona because the symptoms were mild, not like what people say. But I was sure when my sleep became strange, as if I woke up not asleep, and after that I fell asleep for an hour or two, and sometimes I did not sleep. .	1	0	1	0	0	0	1	0	0	0	0	1

^a^We labeled each tweet with 1 or 0 to indicate Arabic Infectious Diseases Ontology classes.

^b^We labeled each tweet as 1 if the person who wrote the tweet was infected and 0 if not.

### Interrater Reliability

We used the Krippendorff alpha coefficient statistic, which supports multilabel input, to test the robustness of the classification scheme for both data sets [32]. The result showed that the Krippendorff alpha score was 0.84 in the influenza data set and 0.91 in the COVID-19 data set, which indicates strong agreement between the two manual coders. The remaining disagreement between the annotators was due to informal terms and Arabic dialects found in social media. For instance, 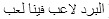 can be understood as “cold is playing with us,” which represents that an uninfected person or flu is playing with us (indicating an infected person). Another example is 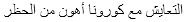, which in English means “get along with Corona is easier than the lockdown.” This may be classified as an infected person or an uninfected person because the word 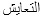 has various meanings.

## Methods

### Overview

In order to create methods to find individuals who have been self-identified as infected and to determine their geolocation in the Twitter data set, we applied multiple supervised learning algorithms on the labeled data set and used NER on the tweet content.

### Multilabel Classification

**Figure 1:**
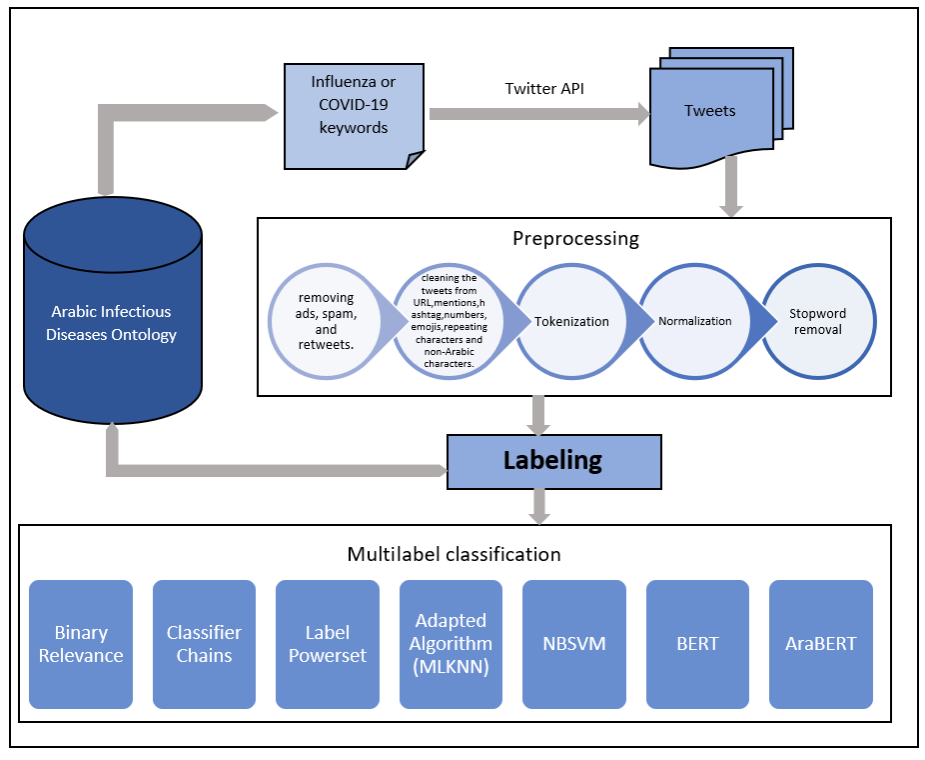
System architecture. API: application programming interface; AraBERT: transformer-based model for Arabic language understanding; BERT: bidirectional encoder representations from transformers; MLKNN: multilabel adapted k-nearest neighbors; NBSVM: support vector machine with naive Bayes features.

The overall architecture of our pipeline for finding infected people is shown in Figure 1. Using a supervised paradigm, we first annotated the corpus with labeling information as described above, before moving on to classify the tweets by applying machine and deep learning algorithms. We used this method for both the influenza and COVID-19 case studies. Each tweet has different labels assigned to it. For instance, the first example in Table 3 contains the labels influenza name (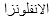), slang term of influenza (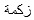), and symptom (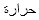). It also represents that the person is infected with influenza. Therefore, we assigned a value of 1 to these labels. On the other hand, the tweet does not include the labels cause, prevention, infection, organ, treatment, diagnosis, place of disease spread, and infected category. Thus, these were marked with 0.

From Table 3, we can see that we have a multilabel classification problem where multiple labels are assigned to each tweet. Basically, the following three methods can be used to solve the problem: problem transformation, adapted algorithm, and ensemble approaches. For each method, there are different techniques that can be used. We applied the following algorithms, which represent ML and deep learning algorithms, to classify the tweets: (1) binary relevance, which treats each label as a separate single class classification problem; (2) classifier chains, which treats each label as a part of a conditioned chain of single-class classification problems, and it is useful to handle the class label relationships; (3) label power set, which transforms the problem into a multiclass problem with one multiclass classifier that is trained on all unique label combinations found in the training data; (4) adapted algorithm (MLKNN), which is a multilabel adapted k-nearest neighbors (KNN) classifier with Bayesian prior corrections; (5) support vector machine with naive Bayes features (NBSVM), which combines generative and discriminant models together by adding NB log-count ratio features to SVM [33]; (6) bidirectional encoder representations from transformers (BERT), which is a condition where all left and right meanings in both layers are used to pretrain deep bidirectional representations from unlabeled text [34]; and (7) transformer-based model for Arabic language understanding (AraBERT), which is a pretrained BERT model designed specifically for the Arabic language [35].

Since some labels were 0 for most tweets, we removed these labels in order to avoid overfitting. In other words, we removed the labels that did not appear in most tweets as shown in Table 3. The remaining important labels were determined depending on the disease case study because they represented different values for different tweets as justified in Table 2. For influenza, they are influenza name, slang term of influenza, symptom, prevention, treatment, and infected with. While for COVID-19, they are name, slang term of COVID-19, symptom, cause, place, and infected with. We also repeated the experiment twice to show the effectiveness of the informal terms in the results. One of them had the labels “disease name,” “slang term of infectious disease,” and “infected with,” and the other had all labels, except “slang term of infectious disease” in both case studies.

In our study, we used the Python scikit-multilearn [36] and ktrain [37] libraries and applied different models. To extract the features from the processed training data, we used a word frequency approach. We split the entire sample into 75% training and 25% testing sets.

### NER

We followed NER systems that used ML algorithms to learn NE tag decisions from annotated text. We used the conditional random fields (CRF) algorithm because it achieved better results than other supervised NER ML techniques in previous studies [29].

**Figure 2:**
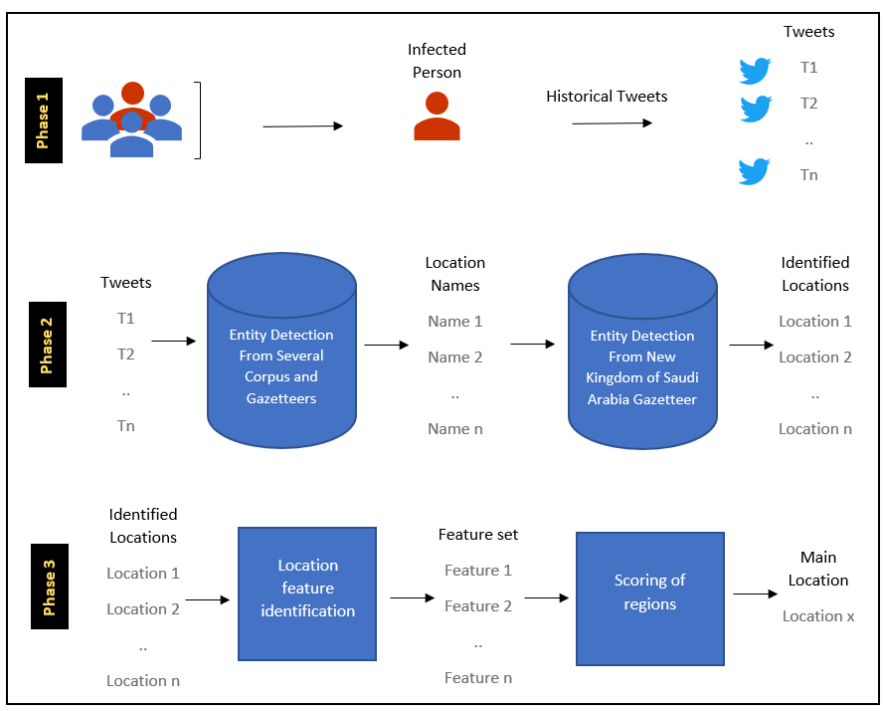
Three phases of the geolocation detection algorithm.

There were three phases in our geolocation detection algorithm as shown in Figure 2. In phase 1, the infected person was specified from the multilabel classification algorithm described in the previous section. Then, we retrieved the historical tweets of this person (around 3000 tweets per person on average) and passed them to the next phase.

Phase 2 consisted of two consecutive stages. First, the tweets were submitted to a named entity detection algorithm to select location records from multiple corpora and gazetteers, including ANERCorp [38,39], and ANERGazet [40]. A set of location names needs to be filtered out from the general names and ambiguous ones. For example, the word 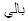 (Bali in English) can be a province in Indonesia or “my mind” as an informal term in Arabic. This step is important in order to ensure that all unrelated location names are not included in the final phase. Second, the identified locations were determined by applying our new entity detection gazetteer, which represents Saudi Arabia regions, cities, and district. The data, which will be released on GitHub [31], are public data collected from the Saudi Post website [41].

In phase 3, common features were identified, such as the most frequent locations, as well as other features, such as occurrence time, which gives a higher score for locations within the last 6 months. Then, each location is scored by a number, which allows us to rank the list and determine the best estimated main location of the user.

After each tweet set with a predictable location, we compared this location with the location field mentioned in the user account, which is not always set by the user because it is an optional field. Here, we kept only users with valuable location information in either the location or description fields.

### Ethical Considerations

Although Twitter has obtained informed consent from users to share information, there was a need to obtain research ethics approval from our university, especially considering our focus on health-related topics [42]. Ethical approval for this study was obtained from Lancaster University on June 21, 2019 [43].

## Results

### Multilabel Classification

A multilabel classification problem is more complex than binary and multiclass classification problems. Therefore, various performance measures were calculated to evaluate the classification process, such as accuracy, F1 score, recall, precision, area under the receiver operating characteristic curve (AUC), and Hamming loss [44]. For all these measures, except Hamming loss, higher scores are better. For Hamming loss, smaller values reflect better performance. It is important to note that the accuracy score function in multilabel classification computes only subset accuracy, which means a sample of labels will be taken in the calculation process, as mentioned previously [36].

Table 4 illustrates the performance measures of the seven models on our training data set with six, five, and three labels for the influenza case study. In the six labels, which are “influenza name,” “slang term of influenza,” “symptom,” “prevention,” “treatment,” and “infected with,” the classifier chains algorithm achieved the highest results in most measures compared with the other algorithms. It had an F1 score of 86.1%, recall of 81.0%, precision of 91.8%, AUC of 88.6%, accuracy of 56.2%, and Hamming loss of 8.9%. The label power set algorithm provided a result slightly lower than the classifier chain by around 2%. The lowest F1 score was observed for NBSVM, which was 58.9%.

The repeated experiment results for the seven models on our training data set with three labels, which were “influenza name,” “slang term of influenza,” and “infected with,” and five labels, which were “influenza name,” “symptom,” “prevention,” “treatment,” and “infected with,” are described in Table 4. There was up to 20% enhancement for accuracy in the seven algorithms. The highest F1 score was achieved by the classifier chains algorithm, which was 88.8%. The recall and precision ranged from 60% to 92%. Consequently, informal terms were shown to represent key factors in the classification process.

Table 5 shows the performance measures of the seven models on our training data set with six, five, and three labels for the COVID-19 case study. Here, the six labels were different from those in the previous case study because they were determined according to the results from the number of tweets in each label as explained in Table 2. The six labels were “COVID-19 name,” “slang term of COVID-19,” “symptom,” “cause,” “place of disease spread,” and “infected with category.” The best results were achieved by the BERT algorithm with an F1 score of 88.2%, recall of 86.7%, precision of 89.7%, AUC of 90.3%, accuracy of 62.0%, and Hamming loss of 8.8%.

The repeated experiment results for the seven models on our training data set with three labels, which were “COVID-19 name,” “slang term of COVID-19,” and “infected with,” and five labels, which were “COVID-19 name,” “symptom,” “cause,” “place of disease spread,” and “infected with category” are described in Table 5. There was up to 20% enhancement for accuracy in the seven algorithms. The highest F1 score was achieved by the BERT algorithm, which was 94.8%, followed by AraBERT, which was 93.3%. The informal terms in the COVID-19 case study showed around 15% enhancement in the evaluation results.

**Table 4:** Training results of the seven algorithms with six, five, and three labels for the influenza case study.

Number of labels and multilabel classification techniques			F1 score (%)		Recall (%)		Precision (%)		AUC^a^ (%)		Accuracy (%)	Hamming loss (%)
**Six^b^**												
	Binary relevance	73.1		74.4		71.9		79.7		39.6		18.7
	Classifier chains	86.1		81.0		91.8		88.6		56.2		8.9
	Label power set	85.7		83.8		87.6		88.7		56.2		9.7
	Adapted algorithm (MLKNN^c^)	76.9		75.5		78.4		82.3		39.9		15.5
	BERT^d^	78.1		83.4		73.4		85.4		38.9		13.7
	AraBERT^e^	79.7		72.7		88.2		83.9		49.2		12.5
	NBSVM^f^	58.9		46.3		81.2		70.9		26.8		18.9
**Five^g^**												
	Binary relevance	75.5		76.9		74.1		80.7		45.1		18.3
	Classifier chains	88.0		85.7		90.5		90.2		64.9		8.5
	Label power set	87.6		86.2		89.2		90.0		63.9		8.9
	Adapted algorithm (MLKNN)	79.9		76.4		83.9		84.0		47.9		14.0
	BERT	84.1		83.1		85.0		88.0		57.5		10.3
	AraBERT	87.3		86.3		88.4		90.0		64.3		9.0
	NBSVM	61.6		49.7		81.2		72.0		26.8		20.2
**Three^h^**												
	Binary relevance	80.8		80.0		81.7		81.2		60.4		18.8
	Classifier chains	88.8		85.7		92.2		89.3		72.4		10.7
	Label power set	88.3		88.0		88.6		88.4		70.8		11.6
	Adapted algorithm (MLKNN)	80.9		84.7		77.5		80.2		54.0		19.8
	BERT	87.6		93.9		82.1		88.9		68.1		11.7
	AraBERT	85.9		81.5		90.9		86.8		66.9		13.1
	NBSVM	79.5		75.1		84.3		82.1		59.9		17.1

^a^AUC: area under the receiver operating characteristic curve.

^b^The six labels are “influenza name,” “slang term of influenza,” “symptom,” “prevention,” “treatment,” and “infected with.”

^c^MLKNN: multilabel adapted k-nearest neighbors.

^d^BERT: bidirectional encoder representations from transformers.

^e^AraBERT: transformer-based model for Arabic language understanding.

^f^NBSVM: support vector machine with naive Bayes features.

^g^The five labels are “influenza name,” “symptom,” “prevention,” “treatment,” and “infected with.”

^h^The three labels are “influenza name,” “slang term of influenza,” and “infected with.”

**Table 5:** Training results of the seven algorithms with six, five, and three labels for the COVID-19 case study.

Number of labels and multilabel classification technique			F1 score (%)		Recall (%)		Precision (%)		AUC^a^ (%)		Accuracy (%)		Hamming loss (%)
**Six^b^**													
	Binary relevance	54.6		52.8		56.6		64.0		15.6		33.3	
	Classifier chains	53.9		49.8		58.7		64.2		18.5		32.3	
	Label power set	58.6		59.4		57.9		66.5		22.2		31.8	
	Adapted algorithm (MLKNN^c^)	54.5		51.0		58.4		64.4		10.0		32.4	
	BERT^d^	88.2		86.7		89.7		90.3		62.0		8.8	
	AraBERT^e^	82.0		84.4		79.8		86.0		50.5		13.6	
	NBSVM^f^	64.3		51.7		85.0		73.1		20.7		21.7	
**Five^g^**													
	Binary relevance	57.0		56.0		58.1		63.1		15.8		35.9	
	Classifier chains	56.2		53.0		59.9		63.3		18.3		35.1	
	Label power set	60.8		63.4		58.4		65.0		22.0		34.8	
	Adapted algorithm (MLKNN)	56.5		54.6		58.7		63.1		10.4		35.7	
	BERT	87.3		87.9		86.7		88.9		59.0		10.9	
	AraBERT	86.3		92.7		80.7		88.6		53.9		12.1	
	NBSVM	55.2		40.6		86.4		67.9		17.9		28.0	
**Three^h^**													
	Binary relevance	68.5		69.0		68.0		69.2		36.9		30.8	
	Classifier chains	69.7		68.1		71.4		71.2		39.9		28.7	
	Label power set	70.3		69.0		71.5		71.6		40.1		28.3	
	Adapted algorithm (MLKNN)	71.6		70.7		72.6		72.8		41.4		27.1	
	BERT	94.8		96.4		93.3		94.9		93.2		5.1	
	AraBERT	93.3		94.8		91.9		93.5		85.3		6.5	
	NBSVM	70.6		59.6		86.5		75.4		46.5		24.2	

^a^AUC: area under the receiver operating characteristic curve.

^b^The six labels are “COVID-19 name,” “slang term of COVID-19,” “symptom,” “cause,” “place of the disease spread,” and “infected with category.”

^c^MLKNN: multilabel adapted k-nearest neighbors.

^d^BERT: bidirectional encoder representations from transformers.

^e^AraBERT: transformer-based model for Arabic language understanding.

^f^NBSVM: support vector machine with naive Bayes features.

^g^The five labels are “COVID-19 name,” “symptom,” “cause,” “place of the disease spread,” and “infected with category.”

^h^The three labels are “COVID-19 name,” “slang term of COVID-19,” and “infected with.”

### NER

A key point to be noted is that our geolocation detection evaluation is based on the location of users where they were tweeting. We filtered tweets that did not have any information in the location field and/or had nonplausible locations, such as moon and space. We created a manually annotated set from the information in the location field in order to demonstrate greater accuracy. This is due to the ambiguous information in the location field that can be detected by hand. For instance, we found some adjectives of the location, like 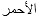

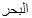

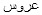
 and 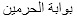, referring to Jeddah city in Saudi Arabia.

In the influenza study, around 907 users were classified as infected with influenza, and 397 of these users provided valuable information in their accounts that could be used to identify the location. As a result, our algorithm achieved an accuracy of 45.8% for predicting locations.

Regarding the COVID-19 study, 915 people were considered to be infected, and around 358 user accounts had useful information about the location. Therefore, after applying the algorithm, the accuracy was up to 63.6% for identifying the locations of the infected users.

## Discussion

### Principal Findings

To understand the effect of deep learning algorithms on the classification process, we needed to compare the results of the ML algorithms with deep learning ones in the two case studies for influenza and COVID-19. In the influenza study, the results of deep learning algorithms and ML ones were close to each other. In other words, there was no improvement in the results when applying deep learning methods, such as BERT and AraBERT. On the other hand, in the COVID-19 case study, there was up to a 25% enhancement in the results when applying BERT and/or AraBERT. These results helped to confirm that deep learning methods show good returns when dealing with new terms or unknown vocabularies that represent COVID-19 terms.

By applying our previous work [45], which classified the sources of the tweets into the following five types: academic, media, government, health professional, and public, we found that informal language was used in the public type (examples 1, 3, and 7 in Table 3), while the other types (academic, media, government, and health professional) utilized more formal styles (examples 2, 4, 5, and 6 in Table 3). Hence, disease-related slang names or other symptoms play an important role in detecting the disease mentions in social media. People not only used slang terms but also expressed their feelings using other terms such as metaphors [46]. For example, “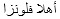,” which means “hi flu,” shows that the person, who wrote the tweet, was affected by flu. Here, 71.9% of the tweets proved that there was a relationship among the informal language used by flu-infected people.

We also found that there was a relationship among the “symptom,” “prevention,” and “infected with” labels. Overall, 64.3% of people infected by influenza sent tweets mentioning symptoms, such as sneezing, headache, coughing, and fever. Among tweets about prevention, 69.3% were written by a person who was not infected with influenza. However, there were a number of tweets that broke these patterns. In other words, we observed tweets written about symptoms that did not represent an infected person or tweets written about prevention that represented an infected person. Table 6 shows some examples of the tweets that described these relationships.

**Table 6:** Examples of tweets describing the relationships among the symptom, prevention, place, and infected with labels.

Tweet in Arabic	Tweet in English	Description
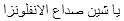	Flu headache is bad	The relationship between symptom and infected with influenza
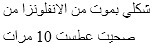	I think I will die from flu; I sneeze 10 times from the time I wake up	The relationship between symptom and infected with influenza
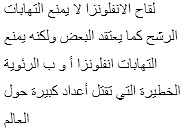	The flu vaccine does not prevent colds, as some believe, but it prevents serious influenza A and B infections that kill large numbers around the world	The relationship between prevention and noninfected with influenza
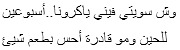	Corona, what did you do for me? For two weeks, I will not be able to feel the taste of something	The relationship between symptom and infected with COVID-19
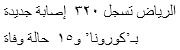	Riyadh records 320 new coronavirus cases and 15 deaths	The relationship between place and noninfected with COVID-19
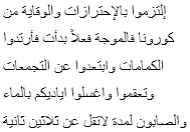	Adhere to the precautions and prevention from Corona, as the wave has really started, so wear masks, stay away from gatherings, and sterilize and wash your hands with soap and water for a period of no less than thirty seconds	The relationship between prevention and noninfected with COVID-19

The study by Saker et al [47], which was published recently, proved that users who tested positive for COVID-19 also reported their symptoms using Twitter. Alanazi et al [23] described the most common COVID-19 symptoms from Arabic tweets in their study. These symptoms can be further evaluated in clinical settings and used in a COVID-19 risk estimate in near real time.

There are many ways to know the location of the Twitter user, such as geocoordinates, place field, user location, and tweet content. The most accurate method is using the network geolocation system for either the tweet or the user. However, because it is an optional field, less than 3% of users provide this information [19,48]. In addition, there is noisy information in the user location field because users can type anything like “home” or “in the heart of my dad.” As a result, we used the tweet content by assuming that users mentioned helpful information when they tweeted.

On the other hand, some researchers have tried to predict the location of the user using dialect identification from the tweet content [49]. Although this may prove fruitful, in our scenario, it may not reflect the current location that would be required, since a person may tweet in the Egyptian dialect but live in Saudi Arabia.

### Conclusion

This paper has, for the first time, shown that Arabic social media data contain a variety of suitable information for monitoring influenza and COVID-19, and crucially, it has improved on previous research methodologies by including informal language and nonstandard terminology from social media, which have been shown to help in filtering unrelated tweets. It should be noted that we are not trying to provide a single source of information for public health bodies to use, but want to provide a comparable information source through which to triangulate and corroborate estimates of disease spread against other more traditional sources.

We also introduced a new Arabic social media data set for analyzing tweets related to influenza and COVID-19. We labeled the tweets for categories in the Arabic Infectious Disease Ontology, which includes nonstandard terminology. Then, we used multilabel classification techniques to replicate the manual classification. The results showed a high F1 score for the classification task and showed how nonstandard terminology and informal language are important in the classification process, with an average improvement of 8.8%. The data set, including tweet IDs, manually assigned labels, and other resources used in this paper, have been released freely for academic research purposes, with a DOI via Lancaster University’s research portal [50].

Moreover, we applied an NER algorithm on the tweet content to determine the location and spread of infection. Although the number of users was limited, the results showed good accuracy in the analysis process.

There are several further directions to enhance the performance of the system in the future, including expanding the data used to train the classifier, analyzing different infectious diseases, and using more NLP techniques and linguistic features.

## Appendix

Multimedia Appendix 1 Guidelines for annotating tweets.

## Abbreviations

API: application programming interface
AraBERT: transformer-based model for Arabic language understanding
AUC: area under the receiver operating characteristic curve
BERT: bidirectional encoder representations from transformers
ML: machine learning
MLKNN: multilabel adapted k-nearest neighbors
NBSVM: support vector machine with naive Bayes features
NER: named entity recognition
NLP: natural language processing
