# Correction: Social Media Monitoring of the COVID-19 Pandemic and Influenza Epidemic With Adaptation for Informal Language in Arabic Twitter Data: Qualitative Study

**DOI:** 10.2196/45742

**Published:** 2023-02-03

**Authors:** Lama Alsudias, Paul Rayson

**Affiliations:** 1 Information Technology Department College of Computer and Information Sciences King Saud University Riyadh Saudi Arabia; 2 School of Computing and Communications Lancaster University Lancaster United Kingdom

In “Social Media Monitoring of the COVID-19 Pandemic and Influenza Epidemic With Adaptation for Informal Language in Arabic Twitter Data: Qualitative Study” (JMIR Med Inform 2021;9(9):e27670) the authors made one addition to the Acknowledgments section and changes to the Corresponding Author address and degree list.

The Acknowledgments has been changed from:

The authors thank Nouran Khallaf, who is a PhD student at Leeds University (mlnak@leeds.ac.uk), for her help in labeling the tweets. LA wishes to thank King Saud University for funding her PhD study.

To:

The authors thank Nouran Khallaf, who is a PhD student at Leeds University (mlnak@leeds.ac.uk), for her help in labeling the tweets. This research project was supported by a grant from the “Research Center of College of Computer and Information Sciences”, Deanship of Scientific Research, King Saud University.

Additionally, the Corresponding Author Address and degree list has been changed from:

Lama Alsudias, BSc, MSc

School of Computing and Communications

Lancaster University

InfoLab21

Lancaster

GB

Phone: 44 1524 510357

Email:l.alsudias@lancaster.ac.uk

To:

Lama Alsudias, BSc, MSc, PhD

Information Technology Department

College of Computer and Information Sciences

King Saud University

Prince Turki Bin Abdulaziz Al Awwal Road

Riyadh, 12371

Saudi Arabia

Phone:: 966 118051044

Email: lalsudias@ksu.edu.sa

The correction will appear in the online version of the paper on the JMIR Publications website on February 3, 2023 together with the publication of this correction notice. Because this was made after submission to PubMed, PubMed Central, and other full-text repositories, the corrected article

