# Supplementary material for: Social Media Monitoring of the COVID-19 Pandemic and Influenza Epidemic With Adaptation for Informal Language in Arabic Twitter Data: Qualitative Study
Source: JMIR Med Inform. 2021 Sep 17;9(9):e27670. doi: 10.2196/27670 (PMC8451962; doi:10.2196/27670)
Supplement: Multimedia Appendix 1 [file medinform_v9i9e27670_app1.docx]

**Annotation Process**

The process is to label them in order to monitor the spread of Influenza. There are 12 classes, ‎‎11 originating from the Arabic Infectious Disease Ontology which are: Infectious disease name, ‎i.e. Influenza or COVID-19 in our case, Slang term of infectious disease, Symptom, Cause, ‎Prevention, Infection, Organ, Treatment, Diagnosis, Place of the disease spread, and Infected ‎category and one new class which is `Infected with', which represents if the user is infected ‎with Influenza or COVID-19. Each tweet is labelled with 1 if the class is included in it or 0 if not ‎included. Moreover, the tweet can be labelled multiple times if it contains multiple classes. For ‎each class described below there is a small description with an example that belong to ‎Influenza case study.‎

Infectious disease name: The tweet is labelled with 1 if it includes the Influenza name in it e.g. ‎‎"‎حملة تطعيم الانفلونزا تبدأ اليوم‎ " in English, ``The flu vaccination campaign starts today''.‎

Slang term of infectious disease: The tweet is labelled with 1 if it contains one of the slang ‎terms of infectious disease which are: " ‎فلو، النزلة، فلونزا، البرد‎ " in Influenza case study and ‎‏"كرونا"‏‎ ‎in the COVID-19 one e.g. " ‎الفلونزا لاعبة فيني لعب‎ " in English, ``Flu is playing with me''.‎

Symptom: The tweet is labelled with 1 when it refers to any Symptom of Influenza or COVID-19 ‎which are high fever, headache, cough, runny nose, muscle pain e.g." ‎مو قادرة اتحرك من قوة الحرارة‎ ‎‎"in English, ‎‏"‏Cannot move because high fever''.‎

Cause: The tweet is labelled with 1 if it mentions the virus which is the cause of infectious ‎disease which are virus in both cases e.g." ‎فيروس الانفلونزا وطرق الوقاية منه‎ "in English, ‎‏"‏Influenza ‎virus and ways to prevent it''.‎

Prevention: The tweet is labelled with 1 when it contains one of the preventative methods ‎which are vaccine, washing hands, and cleaning surfaces e.g." ‎وزارة الصحة توصي بأخذ لقاح الانفلونزا ‏بدءا من اكتوبر‎ " in English, ``The Ministry of Health recommends taking the flu vaccine starting ‎from October''.‎

Infection: The tweet is labelled with 1 if it contains any ways of infection by Influenza or ‎COVID-19 such as cough, sneezing, and touching contaminated surfaces e.g." ‎الطريقة الصحيحة ‏لمنع انتشار رذاذ العطاس بالصور‎ " in English, ``The correct way to prevent the spread of sneeze with ‎pictures''.‎

Organ: The tweet is labelled with 1 when it mentions any part of the body affected by ‎Influenza or COVID-19 such as head or nose e.g. " ‎خشمي صار طماطة من الانفلونزا‎ " in English, ‎‏"‏My ‎nose became tomato from the flu''.‎

Treatment: The tweet is labelled with 1 if it contains one of infectious diseases treatments ‎which are to drink fluids, rest and take sedatives e.g." ‎شرب السوائل يساعد في سرعة الشفاء من الانفلونزا‎ " ‎in English, ``Drinking fluids helps speed recovery from the flu''.‎

Diagnosis: The tweet is labelled with 1 if it includes the method of diagnosis infectious disease ‎which is clinical examination in Influenza and making a test in the COVID-19 one e.g.‎

‎" ‎ساعة ونص انتظار عند الدكتور وبالنهاية بس بنادول. اكره الانفلونزا وقت الاختبارات‎ " in English, ‎‏"‏An hour and a ‎half waiting at the doctor, and at the end, just Panadol. I hate flu in tests time''.‎

Place of the disease spread: The tweet is labelled with 1 if it contains any location e.g.‎

‎" ‎اكاد اجزم انه مكة كلها موبوءة بالفلونزا‎ " in English, ‎‏"‏I can almost confirm that all of regions of Mecca is ‎infected with influenza''.‎

Infected category: The tweet is labelled with 1 if it mentions a category such as kids, old ‎people, or pregnant women e.g. " ‎ماذا تفعل الحامل عند الاصابة بالانفلونزا‎" in English, ‎‏"‏What do ‎pregnant women do when infected with the flu''.‎

Infected with: The tweet is labelled with 1 if it's understood that the user is infected with ‎infectious disease e.g." ‎صداع شديد و الانفلونزا تعبتني‎" in English, ‎‏"‏Severe headache and the flu ‎bored me''.‎
